# Prevalence and correlates of prolonged grief disorder symptom severity in a large sample of Italian adults

**DOI:** 10.1192/bjo.2025.10763

**Published:** 2025-08-01

**Authors:** Alessandro Musetti, Christian Franceschini, Maria C. Quattropani, Holly G. Prigerson, Vittorio Lenzo

**Affiliations:** Department of Humanities, Social Sciences and Cultural Industries, University of Parma, Parma, Italy; Department of Medicine and Surgery, University of Parma, Parma, Italy; Department of Educational Sciences, University of Catania, Catania, Italy; Cornell Center for Research on End-of-Life Care, Weill Cornell Medicine, New York, New York, USA; Department of Radiology and Medicine, Weill Cornell Medicine, New York, New York, USA

**Keywords:** Prolonged grief disorder, suicide, depression, anxiety, stress

## Abstract

**Background:**

Literature has shown that a significant minority of bereaved people are at risk of prolonged grief disorder (PGD). However, studies on its prevalence and correlates within Italian samples remain scarce.

**Aims:**

This study aimed to explore the prevalence and correlates of PGD symptom severity among 1603 bereaved Italian adults.

**Method:**

Self-reported data on PGD, suicidal ideation, depression, anxiety and stress were gathered. Descriptive characteristics and bereavement-related information were also collected.

**Results:**

Among participants who lost a close other person at least 12 months prior, the prevalence of probable PGD and severe suicidal ideation was 7.7% (*n* = 104) and 0.7% (*n* = 9), respectively. The overall prevalence of severe suicidal ideation in the sample was 4.5%, rising to 18.2% among those with probable PGD. The probable PGD diagnosis showed minimal agreement with reported depression (phi = 0.25), anxiety (phi = 0.19), and stress (phi = 0.26), suggesting potentially limited overlap and supporting their distinctiveness. The severity of PGD symptoms was significantly positively associated with older age and suicidal ideation, and negatively associated with lower educational background and time since loss. PGD severity also varied by kinship, cause of death and place of residence. Specifically, bereaved individuals who lost a grandparent due to natural causes associated with ageing and lived in small- to medium-sized cities reported lower PGD symptom severity relative to others.

**Conclusions:**

These findings contribute to the understanding of PGD symptomatology in bereaved individuals in Italy, although the results may not generalise to the entire Italian population.

The death of a loved one, while common, is nonetheless an upsetting and disruptive experience. In fact, it has been rated as one of the most stressful life events.^1^ While grief reactions can vary, in cases of natural death the typical response primarily involves acceptance of the death and yearning for the deceased. Negative indicators of grief, such as disbelief, yearning, anger and depression, typically decrease around 6 months post-loss, with most individuals experiencing reduced symptoms by 12 months.^2^ Nevertheless, some individuals experience a chronically severe and disabling grief reaction known as prolonged grief disorder (PGD). In 2022, PGD was added to DSM-5-TR.^3^ The criteria for PGD include a deep yearning or longing for the deceased loved one, along with a preoccupation with thoughts or memories of them. Additionally, other cognitive, emotional and behavioral symptoms – such as a sense of disbelief regarding the death, detachment from others and disruption of identity – are required for a PGD diagnosis. According to the DSM-5-TR, to differentiate PGD from normal grief, symptoms must be severe, persist for at least 12 months after the death and be associated with significant functional impairment.

The growing interest in grief research stems from its impact on the medical and psychological health of the bereaved. Although there is heterogeneity across studies, findings converged in indicating that grief is related to an increased risk of mortality, especially in the first months after loss.^4^ Suicidal thoughts or behaviours may also be present, even after controlling for the effect of other mental disorders, such as major depression.^3,5^ Regarding the epidemiology of PGD, previous studies have found a pooled prevalence of 9.8% (95% CI 6.8–14.0), with various factors influencing this percentage. A more recent study based on the DSM-5-TR^3^ criteria has found analogous findings, with prevalence ranging from 4 to 15%.^6^ Also, a German study has found a prevalence rate of 3.3%,^7^ while this percentage was higher (4.2%) when considering the ICD-11 criteria.^8^ Although research is emerging elsewhere,^9,10^ most of these studies on grief were conducted in Western countries. Understanding grief outcomes across different cultural contexts is important as cultural norms and social support systems may influence the expression of PGD symptoms.

Because of the healthcare costs of bereavement, understanding the prevalence of PGD, as well as cross-cultural comparisons, is paramount. To date, surprisingly, studies conducted on the Italian population have been limited to special samples, such as caregivers of terminally ill patients or those in a vegetative state.^11–14^ Therefore, conducting research on the general population is essential. Findings from these studies revealed that the prevalence of PGD ranges from 4.6 to 15%, often co-occurring with symptoms of depression.

The differential diagnosis of PGD with depressive disorders has received great attention in the literature. Since the initial debate on its introduction as a possible new diagnostic category, research has investigated the relationship between PGD and other bereavement-related psychopathology, firstly depression.^5,15–18^ Although PGD may resemble other mental disorders, there is evidence of its distinctiveness.^6^ Nevertheless, comorbidity with depressive disorders and other symptoms may frequently occur. A recent meta-analysis has found that severe PGD symptoms co-occur with severe depression, anxiety and post-traumatic stress symptoms in 63, 54 and 49% of cases, respectively.^19^ Moreover, the co-occurrence of other mental disorders can influence cognitive capacity among the bereaved. Concerning possible cognitive difficulties associated with bereavement, Fisher and colleagues^20^ found that the co-occurrence of PGD with depression and anxiety was strongly related to perceived cognitive failures in a sample of 581 bereaved participants. Accordingly, investigating depression and other frequent symptoms associated with bereavement is fundamental for understanding its overall impact.^10^ Recently, PGD has been recognised as primarily linked to dysfunction in the reward system.^21^ However, examining stress is crucial for understanding how acute stress responses may interact with these deficits – such as those in the regulation of the reward system – to exacerbate or prolong the grieving process. To date, though, little is known about the co-occurrence of severe PGD symptoms with high levels of depression, anxiety and stress in Italy.

As one way to grasp the large variation in grief outcomes between individuals, several studies have examined the role of demographic factors and aspects related to the loss in predicting PGD symptoms. A systematic review of studies published from 1994 to 2016 found older age was associated with higher severity of symptoms.^22^ Also, there are marked differences between men and women in how to confront loss,^23,24^ though research on the relationship between grief and gender has found inconsistent results.^9^ Several studies have also examined factors such as time since the loss, kinship relationship to the deceased, cause of death and the condition of being the main caregiver in relation to bereavement. In a study involving a sample of 546 bereaved emerging adults, Schwartz and colleagues found that time since the loss had a significant effect on PGD symptoms (the more recent the loss, the higher the severity of symptoms).^25^ However, other studies found no significant relationships.^26^ Once again, no evidence is available regarding the Italian context.

The first aim of this study was to explore the prevalence rate of probable cases of DSM-5-TR-based PGD and suicidal ideation in a large sample of Italian adults. Following the DSM-5-TR criterion A, we investigated the rate of PGD among individuals who lost a loved one at least 12 months ago. For exploratory purposes, we also examined the prevalence rate among bereaved individuals who lost a loved one less than 12 months ago. The second aim was to explore the associations between PGD symptoms and depression, anxiety and stress, as well as to investigate PGD’s distinctiveness from other common mental disorders secondary to bereavement. The third aim was to identify correlates associated with the severity of PGD symptoms and cases of PGD.

## Method

### Participants

A total of 1716 participants agreed to take part in this cross-sectional study, with 1663 completing the study (97% participation rate). Forty cases with missing or incomplete data (cases with more than 5% of responses missing) were excluded from the analyses. An additional 20 cases were identified as extreme values based on the variable ‘time since loss’, a key factor in grief research. Specifically, these cases had values exceeding 1.5 times the interquartile range (IQR) from the median, indicating extreme deviations from the normal distribution of this variable. The mean time from loss for the excluded subjects was 41.42 years. Therefore, the final sample comprised 1603 participants, recruited through convenience sampling. Participation in the study was voluntary, and no form of compensation was provided to participants.

### Procedure

Participants were recruited through university communication systems, social networks, online blogs and other similar channels (e.g. WeChat groups). Data were collected via an anonymous online survey. Informed consent was obtained electronically through a consent form, which participants had to agree to before proceeding with the survey. This process ensured that participants were fully informed about the nature of the study and their rights, including the right to withdraw from the research. The survey took approximately 20 min to complete. Eligible participants were required to be at least 18 years old and to have experienced the loss of a loved one prior to participating in the survey. Participants were excluded if they did not meet the age criterion or if the time since loss was identified as an outlier based on the IQR.

### Measures

Demographic information collected included age, gender, educational background and place of residence. Bereavement-related information comprised time since the loss, kinship relationship to the deceased, cause of death and whether the participant was the main caregiver.

Suicidal ideation was assessed using item 9 of the Beck Depression Inventory–II (BDI-II),^27,28^ which is primarily designed for screening purposes in research settings rather than as a definitive measure of suicidal thoughts or for estimating prevalence at the population level. Responses to this item were categorised as follows: 0 = ‘I don’t have any thoughts of killing myself’, 1 = ‘I have thoughts of killing myself, but I would not carry them out’, 2 = ‘I would like to kill myself’ and 3 = ‘I would kill myself if I had the chance.’ These responses were analysed as categories rather than as a continuous variable.

The Prolonged Grief Questionnaire-13 (PG-13)^18^ is a self-report instrument for assessing the potential PGD and the severity of reported symptoms according to the DSM-5 and ICD-11 criteria. In this study, we utilised the Italian version of the PG-13, validated by De Luca and colleagues^29^ but based on the older DSM-5 criteria. Although the PG-13 has recently been revised (PG-13-R),^6^ an official Italian version of this updated instrument has not yet been released. We modified PG-13 to reflect the DSM-5-TR diagnostic criteria for PGD, allowing us to identify potential cases of PGD and to obtain a score for PGD symptoms severity (i.e. questions Q3 to Q12 of the PG-13-R). Additionally, we included an item on social support to assess ‘intense loneliness as a result of the death’, as indicated by criterion C of PGD.^3^ Respondents rated each item on a 5-point Likert scale ranging from ‘1’ (‘not at all’) to ‘5’ (‘overwhelmingly’), yielding total scores for symptom severity ranging from ‘10’ to ‘50’, where higher scores indicate greater intensity. To investigate PGD prevalence, we relied on criteria grounded in the DSM-5-TR^3^ that were included in the PG-13-R.^6^ A diagnostic algorithm reflecting these criteria was employed to identify potential PGD cases.^6^ Specifically, we considered individuals who responded ‘yes’ to three gatekeeper questions regarding the loss of a significant person (Q1 of the PG-13-R), the time elapsed since the death (Q2 of the PG-13-R) and significant impairment associated with symptoms (Q3 of the PG-13-R). Additionally, we looked for scores of 4 or 5 in items Q3 (yearning) and/or Q4 (preoccupation), as well as in at least three of the eight items outlining symptoms defined in both DSM-5-TR criterion C and the PG-13-R.^3,6^ The internal consistency for this sample was excellent, with a Cronbach’s α of 0.87 for the ten items rated on the Likert scale.

The Italian version of the Depression Anxiety Stress Scale – 21 (DASS-21)^30^ is a self-report instrument for measuring depression, anxiety and stress. It consists of 21 items rated on a 4-point Likert scale, ranging from ‘never’ (0) to ‘always’ (3). Items are clustered into three subscales as follows: depression (DASS-21 Depression), assessing dysphoria, self-deprecation, anhedonia, devaluation of life and hopelessness; anxiety (DASS-21 Anxiety), including autonomic arousal, skeletal muscle effects and subjective experience; stress (DASS-21 Stress), evaluating irritability, impatience, difficulty relaxing and nervous arousal. Scores for each subscale can be calculated by summing the scores for the relevant items. For the Italian version,^31^ the average cut-off scores are established at 3.5 for depression, 2.4 for anxiety and 6.4 for stress. In this study, the Italian version of the DASS-21^31^ demonstrated excellent psychometric properties. The degree of internal consistency of this sample was also excellent for all three subscales, with a Cronbach’s *α* of 0.91 for depression, 0.86 for anxiety and 0.89 for stress.

### Data analysis

Data were analysed using SPSS version 29 for Windows (IBM, Armonk, NY, USA). Descriptive statistics are reported as frequencies (%), mean scores and standard deviations. Extreme values for the time-since-loss variable were identified using the boxplot method, based on Tukey’s criterion,^32^ which defines outliers as values exceeding 1.5 times the IQR from the first or third quartile. To assess the robustness of the findings, a sensitivity analysis was conducted including these outliers, and the results remained substantially unchanged. Phi coefficients were calculated to assess associations between the potential diagnosis of PGD and emotional states of depression, anxiety and stress. Pearson product-moment correlation analysis was employed to explore the relationships between the severity of PGD symptoms score, the demographic and bereavement-related information, suicidal ideation, DASS-21 Depression, Anxiety and Stress. Independent *t*-tests, analysis of variance (ANOVA) with post hoc evaluation using the Bonferroni technique and χ² test of association were used to examine correlates of the severity of PGD symptoms.

## Results

### Characteristics of the participants

Table 1 shows the demographic and bereavement-related information of the sample. The final sample consisted of 1603 participants, aged 18 to 91 years (mean 29.39, s.d. = 13.27). Most participants were female (62.1%, *n* = 995), had a high school diploma (63.3%, *n* = 1015) and lived in a medium-sized city (48.5%, *n* = 771). Among the participants, 50.9% (*n* = 816) were grandchildren of the deceased, and 15.8% (*n* = 253) were the main caregivers. The average time since the loss was 72.87 months (s.d. = 74 months), with 65.3% (*n* = 1046) reporting that their loved one died from a chronic illness (e.g. cancer, dementia, neurodegenerative disorder).

**Table 1:** Demographic and bereavement-related information (*n* = 1603)

Variables	Mean (s.d.) (*n*, %)
Age of participants (years)	29.39 (13.27)
Gender	
Male	608 (37.9)
Female	995 (62.1)
Educational background	
Primary or middle school diploma	140 (8.7)
High school diploma	1015 (63.3)
Graduate	395 (24.6)
Postgraduate	53 (3.3)
Place of residence	
Small city (<50,000 inhabitants)	630 (39.6)
Medium city (50,000–250,000 inhabitants)	771 (48.5)
Large city (>250,000 inhabitants)	190 (11.9)
Time since loss (months)	72.87 (74.00)
Kinship to deceased	
Grandchild	816 (50.9)
Son/daughter	332 (20.7)
Sibling	32 (2.0)
Spouse	29 (1.8)
Child	17 (1.1)
Other	377 (23.5)
Cause of death	
Illness	1046 (65.3)
Natural causes associated with ageing	328 (20.5)
Accidents	179 (11.2)
Suicide	29 (1.8)
Other	18 (1.1)
Main caregiver	
Yes	253 (15.8)
No	1350 (84.2)

There are 12 and 3 missing data for place of residence and cause of death, respectively.

### Prevalence of PGD symptoms, perceived support and suicidal ideation

The mean score for the total PG-13 was 21.32 (s.d. = 7.47), with a range of 10–49. Among those who lost a close person at least 12 months prior (*n* = 1355), the prevalence of individuals potentially at risk of PGD according to DSM-5-TR criteria was 7.7% (*n* = 104). The symptom of disbelief about the death had the highest mean score (mean 3.07, s.d. = 1.16), while difficulties with reintegrating into relationships with others and activities after the death had the lowest (mean 1.69, s.d. = 1.01). The mean score for item 9 of the BDI-II, which assesses suicidal ideation, was 0.18 (s.d. = 0.51). Among the participants, 0.7% (*n* = 9) reported ‘I would kill myself if I had the chance’, while 3.8% (*n* = 51) expressed ‘I would like to kill myself’. Among those potentially at risk of prolonged grief (*n* = 104), 1.9% (*n* = 2) indicated ‘I would kill myself if I had the chance’, and 16.3% (*n* = 17) stated ‘I would like to kill myself’.

### Prevalence of depression, anxiety and stress and relationships with PGD symptoms

The prevalence of moderate to extremely severe levels of depression, anxiety and stress in the entire sample was 47.4% (*n* = 759), 47% (*n* = 753) and 35.9% (*n* = 576), respectively. Mean scores were 6.74 (s.d. = 5.11) for depression, 4.94 (s.d. = 4.26) for anxiety and 8.28 (s.d. = 4.62) for stress, all exceeding the established cut-off scores.^31^ Among participants who lost a loved one at least 12 months prior (*n* = 1355), the potential PGD diagnosis showed modest agreement with depression (phi = 0.25), anxiety (phi = 0.19) and stress (phi = 0.26), suggesting limited overlap. Correlation coefficients between the severity of PGD symptoms and DASS 21 scores for depression, anxiety and stress were 0.59, 0.49 and 0.59, respectively (*p* < 0.001), indicating significantly positive associations.

### Correlates of PGD symptom severity

With regard to correlates of the severity of PGD symptoms in the total sample, females (mean 22.39 ± 8.02) compared with males (mean 19.57, *SD* = 6.08) showed significantly higher scores (*t*(1601) = −7.46, *p* < 0.001; Cohen’s *d* = 0.38). Participants who were the main caregiver (mean 26.16 ± 8.70) reported higher symptom severity than those (mean 20.41 ± 6.85) who were not (*t*(1601) = 11.71, *p* < 0.001; Cohen’s *d* = 0.80). The severity of PGD symptoms was positively associated with age (*r* = 0.12, *p* < 0.001), indicating older age was associated with higher PGD severity of symptoms. Conversely, educational background was negatively correlated (*r* = −0.08, *p* < 0.001), suggesting lower education levels are associated with higher severity. Furthermore, the severity of PGD symptoms negatively correlated with time since the loss (*r* = −0.14, *p* < 0.001). This means that a longer time since the loss is associated with lower PGD severity. It was also positively correlated with suicidal ideation (*r* = 0.29, *p* < 0.001), indicating that higher suicidal ideation is related to greater severity of PGD symptoms. All correlation coefficients were highly statistically significant but with magnitudes below 0.30, reflecting modest associations. The severity of PGD scores differed significantly by kinship (*F*(5, 1597) = [46.84], *p* < 0.001; *η* ^2^ = 0.13), cause of death (*F*(4, 1595) = [15.84], *p* < 0.001; *η^2^* = 0.04) and place of residence (*F*(2, 1588) = [5.06], *p* = 0.006; *η* ^2^ = 0.01). Results of the Bonferroni post hoc test indicated that the severity of PGD symptom scores was lower when the bereaved person was a grandchild compared with all other kinships (*p* < 0.001). The Bonferroni post hoc test for multiple comparisons also revealed that the mean scores were significantly higher when the lost person was a child, a sibling or a spouse, compared with a grandparent, parent or other kinship to the deceased (*p* < 0.001). Participants who lost a loved one for natural causes associated with ageing scored significantly lower than for illness (*p* < 0.001), accidents (*p* < 0.001), suicide (*p* = 0.001) or other causes of death (*p* = 0.011). The Bonferroni post hoc test also showed that participants living in large cities scored significantly higher than those living in medium (*p* = 0.029) and small cities (*p* = 0.005). No statistical differences were found between the mean scores of people living in small and medium cities.

We further investigated factors associated with probable cases of PGD among those bereaved at least 12 months earlier (*n* = 1355). Participants potentially at risk of PGD differed in age (*t*(1353) = −5.37, *p* < 0.001; Cohen’s *d* = 0.55). Older age was associated with cases who met the criteria (36.61 ± 15.88 *v*. 29.24 ± 13.23, respectively). Potential cases of PGD also differed for gender (*χ* ^2^(1) = 19.84, *p* < 0.001), educational background (*χ* ^2^(3) = 15.58, *p* = 0.001), caregiver status (*χ* ^2^(1) = 38.07, *p* < 0.001), place of residence (*χ* ^2^(2) = 14.77, *p* < 0.001), kinship to the deceased (*χ* ^2^(5) = 170.60, *p* < 0.001) and cause of death (*χ* ^2^(4) = 23.07, *p* < 0.001). Specifically, older participants, women and those with only a primary or middle school diploma were more represented among potential PGD cases. The condition of being the main caregiver, living in a large city and having lost a child, as well as suicide as cause of death, were further correlates associated with a higher likelihood of potential cases of PGD.

## Discussion

The prevalence of PGD in adult bereavement has been investigated in many countries, including the UK, the USA and the Netherlands.^6^ While the new DSM-5-TR criteria are now available,^3^ most of the current research still relies on the DSM-5 criteria.^33^ Furthermore, there is still a notable lack of studies specific to the Italian context. In the present study, using the DSM-5-TR^3^ diagnostic criteria, the prevalence of probable PGD in a large Italian sample was 7.7%. This sample is not representative of the entire Italian population, limiting generalisability, and reflects a younger demographic, which may not capture certain types of losses, such as those involving a child or spouse. Nonetheless, this prevalence is slightly lower than the 10% reported in Lundorff and colleagues’ review.^22^ When considering studies that focus on younger samples, He and colleagues^9^ found a significantly lower prevalence of 1.8% (8 cases out of 445) in their sample. However, it is important to note that 35% of our sample was older than the typical university age in Italy (18–26 years), despite the mean ages being quite similar (27.60 years in He et al and 29.39 years in our study). This suggests that differences in sample composition, such as a higher proportion of older participants in our study, might partly account for the higher prevalence we found.

In terms of cross-cultural comparisons, Prigerson and colleagues^6^ reported PGD prevalence of 6.3, 16.6 and 11.3% for the US, Netherlands and UK samples, respectively. Our findings are higher than the US sample but lower than the other two. Similarly, Boelen and Lenferink found a 10.1% prevalence, slightly higher than ours.^26^ These variations underscore the importance of considering factors such as diagnostic criteria changes, cultural differences, sample characteristics and assessment tools when interpreting prevalence rates.^34^ More research, particularly in the Italian context, is still needed, though this study aims to contribute to closing this gap.

Regarding cross-cultural comparisons, studies on PGD symptoms in Far Eastern cultures have produced mixed results. For instance, our sample showed a lower prevalence of symptoms (7.7%) compared with the findings of Li and Prigerson (13.9%).^10^ In the Italian context, our sample exhibited more intense symptoms than those reported by Sardella and colleagues.^14^ However, key demographic differences between samples must be noted. Sardella and colleagues^14^ focused on bereavement due to cancer, while our sample included a broader range of causes of death and a longer time since the loss. These differences may partly explain the discrepancies in prevalence rates. Furthermore, many studies still use the DSM-5 criteria for Persistent and Complex Bereavement Disorder,^33^ which may contribute to varying prevalence figures.

A key finding in our study was the prevalence of suicidal ideation, a symptom increasingly recognised in the literature on bereavement.^4^ Suicidal ideation prevalence ranges from 9 to 49% depending on the cause of death.^35^ In our sample, the pooled prevalence of severe suicidal ideation was 4.5%, rising to 18.2% among those with probable PGD. Freud, in his classic work ‘Mourning and melancholia’, suggested that suicide is more often linked to depression than to mourning itself.^36^ More recent research, such as Sekowski and Prigerson, has confirmed that suicidal ideation is associated with PGD severity symptoms as well as depression.^37^ Our findings emphasise the importance of assessing suicide risk in individuals with PGD, although the cross-sectional nature of the present study precludes determining any causal relationship. In addition, the use of a single-item measure from the Beck Depression Inventory-II (BDI-II), utilised for research purposes rather than diagnostic evaluation, necessitates caution in interpreting the complexity and intensity of suicidal ideation. Future research using clinical interviews will be essential to better understand this risk, as self-report measures tend to overestimate the prevalence of mental health disorders.^38^

The second aim of this study was to examine the co-occurrence of PGD symptoms with depression, anxiety and stress. Consistent with previous research showing that 70% of PGD cases co-occur with symptoms of other mental disorders,^19^ our findings revealed that depression and anxiety were present in about half of the cases, with stress affecting about one-third. The average scores for these measures exceeded the established cut-off points for the Italian population,^31^ suggesting a significant consistency with clinical samples. Several hypotheses could explain this overlap, including the possibility that PGD contributes to the development of depression, anxiety or post-traumatic stress symptoms. However, understanding the causal relationships between these conditions is critical, as they may influence each other in complex ways. While PGD frequently co-occurs with other disorders, it is important to acknowledge its unique diagnostic features. According to the DSM-5-TR,^3^ PGD is recognised as a distinct mental health diagnosis, with specific criteria that differentiate it from major depressive disorder and post-traumatic stress disorder. Research by Prigerson and colleagues has highlighted some degree of distinction between PGD and these disorders.^6^ Similarly, in our study, the weak associations observed between PGD and depression, anxiety and stress (all phi < 0.30) suggest limited overlap, although further research is needed to confirm this distinction more robustly.

The last aim was to explore correlates of PGD symptom severity. In this regard, we conducted analyses to examine demographic and loss-related characteristics correlating with worsening grief symptoms among a sample of 1603 adults, including 1355 individuals who lost a loved one at least 12 months earlier. Findings from this analysis enhance our understanding of the correlates of PGD severity, including demographic and bereavement-related characteristics associated with the severity of PGD symptoms. Specifically, being female, older, having a low educational background and being the main caregiver were significantly related to higher reported symptoms. However, while highly statistically significant, these associations were modest in strength (all correlation coefficients < 0.30), suggesting that while these factors are related to PGD symptom severity, their role appears to be limited. These findings align with existing literature, which often reports weak relationships, and although not all demographic characteristics were consistently associated with PGD severity, the results highlight the complexity of these associations. For instance, a recent systematic review and meta-analysis indicated that both female gender and lower education levels are statistically associated with PGD occurrence.^39^ Similarly, a longitudinal study based on the new DSM-5-TR criteria found lower education to be a significant correlate of PGD.^26^ Age plays a significant role in the experience of grief, with evidence suggesting that older individuals may exhibit a higher occurrence of PGD symptoms. Lundorff and colleagues noted this correlation,^22^ although the relationship between age and grief responses can be complex and nonlinear, as highlighted by Buur and colleagues, who did not find a similar association.^39^ The role of being the main caregiver was also associated with more severe PGD outcomes. Although symptoms typically decrease over time, it is important for practitioners to recognise the potential risk of PGD among caregivers. Furthermore, our study revealed that the severity of PGD symptoms varies based on kinship to the deceased and the cause of death. For instance, in cases of natural causes of death associated with ageing, bereaved individuals reported similar symptoms, albeit with less intensity. This finding was echoed by Buur et al, who also found that unexpected or violent deaths significantly impacted PGD severity.^39^

Forthcoming research should address several limitations of this study. First, the reliance on self-report instruments to assess depression, anxiety, stress and suicidal ideation, while using validated scales such as DASS-21 and BDI-II, may introduce bias. Specifically, item 9 of the BDI-II, used to assess suicidal ideation for research purposes, served as a screening measure rather than a diagnostic evaluation or a prevalence estimate.^40^ Second, although the sample is not representative of the general population and cannot provide reliable population-level prevalence estimates, it could contribute to deepening our understanding of PGD within the Italian context, which remains under-represented in the literature. The oversampling of certain characteristics (e.g. female gender) and the cross-sectional design may further limit the generalisability of the findings – an issue frequently encountered in PGD studies. Moreover, the predominance of university students in the sample – probably due to online convenience sampling – and their limited exposure to certain types of loss, such as the loss of a spouse or a child, may further limit the generalisability of the findings to the broader bereaved population. Lastly, while we utilised a revised version of the PG-13 reflecting DSM-5-TR criteria for PGD, further validation studies are necessary to confirm its effectiveness in diverse populations.

In sum, our findings indicate that many individuals in our sample are at risk of PGD, which increases suicidal ideation. Probable PGD appears distinct from depression, anxiety and stress, with symptom severity associated with demographics (e.g. older age, female gender), bereavement-related factors (e.g. short time since loss, being the primary caregiver) and suicidal ideation. As one of the first studies on PGD in Italy, this research provides valuable insights, though its generalisability is limited by convenience sampling and online recruitment. Despite these limitations, our findings may enhance the understanding of PGD and serve as a starting point for future studies.

## Data Availability

The data that support the findings of this study are available from the corresponding author, V.L., upon reasonable request.
